# An association study between CHEK2 gene mutations and susceptibility to breast cancer

**DOI:** 10.1007/s00580-017-2455-x

**Published:** 2017-04-08

**Authors:** Manizheh Jalilvand, Mana Oloomi, Reza Najafipour, Safar Ali Alizadeh, Najmaldin Saki, Fatemeh Samiee Rad, Mohammad Shekari

**Affiliations:** 1 Department of Medical Genetics, Faculty of Medicine, Hormozgan University of Medical Sciences, Bandar-Abbas, Iran; 2 Department of Molecular Biology, Pasture Institute , Tehran, Iran; 3 Department of Biochemistry and Genetics, Cellular and Molecular Research Center, Qazvin University of Medical Sciences, Qazvin, Iran; 4 Health Research Institute, Thalassemia and Hemoglobinopathy Research Center, Ahvaz Jundishapur University of Medical Sciences, Ahvaz, Iran; 5 Department of Pathology, Metabolic Disease Research Center, Qazvin University of Medical Sciences, Qazvin, Iran

**Keywords:** Breast cancer, CHEK2, Mutation

## Abstract

CHEK2 gene is known as a tumor suppressor gene in breast cancer (BC), which plays a role in DNA repair. The germ line mutations in CEHK2 have been associated with different types of cancer. The present study was aimed at studying the association between CHEK2 mutations and BC. Peripheral blood was collected from patients into a test tube containing EDTA, and DNA was extracted from blood samples. Then, we analyzed mutations including 1100delc, IVS2+1>A, del5395bp, and I157T within CHEK2 gene in patients with BC and 100 normal healthy controls according to PCR-RFLP, allelic specific PCR, and multiplex-PCR. Although IVS2+1G>A mutation within CHEK2 gene was found in two BC patients, other defined mutants were not detected. For the first time, we identified CHEK2 IVS2+1G>A mutation, one out of four different CHEK2 alterations in two Iranian BC patients (2%). Also, our results showed that CHEK2 1100elC, del5395bp, and I157T mutations are not associated with genetic susceptibility for BC among Iranian population.

## Introduction

Breast cancer (BC) is the most common cancer in women. According to WHO statistics, one out of every 8 to 10 women will be diagnosed with BC. In Iran, the risk of BC is one out of every 10 to 15 women with an onset age at least one decade sooner than that observed in developed countries (Noori & Tabarestani 2010). Considering the previous studies, mean age of patients with BC in Iran is 48 years, compared to 55 in other countries (Dvarnia et al. 2012). The genetic factors presumed to be involved in the development and increased risks of BC have been demonstrated in several earlier studies. In some cases, the inheritance of a mutated gene occurs. BC is a severely heterogeneous disease caused by the interaction of genetic and environmental factors, mostly in sporadic cases (Keshavarzi et al. 2011). In some BC cases, the tumor suppressor genes responsible for genome maintenance and DNA repair show a high degree of genomic instability (Noori & Tabarestani 2010).

Defective repair of DNA double-stranded breaks is associated with genetic susceptibility to BC (Noori & Tabarestani 2010; Kilpivaara et al. 2004). Ataxia Telangiectasia Mutated (ATM) is a fragment of BRAC1-associated genome surveillance complex (BASC) and is a primary repair-sensing mechanism. In response to double-strand break repair, checkpoint kinase 2 (CHEK2) is phosphorylated and spontaneously activated by ATM kinases. Later, activated CHEK2 monomers are activated and phosphorylate a large number of substrates, including tumor suppressor upstream gene P53, cell division cycle (Cdc25) family, proteins of serine 988 BRCA1 genes, and cell cycle control proteins (Strachan & Read 1996; Meijers-Heijboer et al. 2002; Bayram et al. 2012). Thus, CHEK2 as a tumor suppressor gene plays an important role in DNA repair and maintenance of chromosomal stability (Nevanlinna & Bartek 2006; Mohelnikova-Duchonova et al. 2010). The CHEK2 gene on chromosome 22q12.1 is prone to mutate and produce cancer (Bayram et al. 2012; Nevanlinna & Bartek 2006).

Despite many studies concerning the association between mutations in CHEK2 gene and the risk of BC around the world to clarify the mechanism of this association, further large-scale in-depth studies still seem to be necessary (Cybulski et al. 2007; Steven & Henry 2007).

In the present study, we focused on four CHEK2 mutations known to affect protein function (c.1100delC, del5395bp, IVS2 + 1G > A, and I175T). The large CHEK2 deletion leads to premature protein truncation at codon 381 (12). The c.1100delC variant also leads to protein truncation at codon 381, making the mutant CHEK2 1100delC protein unstable while abolishing the CHEK2 kinase activity (Cybulski et al. 2006a; Sodha et al. 2006). The splice site mutation (IVS2 + 1G > A) results in a 4-bp insertion due to an abnormal splicing and creates an aberrantly spliced CHEK2 mRNA encoding a truncated protein in exon3 and I157T missense mutation, leading to defective binding to BRCA1, Cdc25A, and p53 (Cybulski et al. 2006b; Bogdanova et al. 2005; Dong et al. 2003). The I157T product (I157T in fork head-associated (FHA) domain of the gene) is a stable protein dimerized with wild-type CHEK2, which is co-expressed in human cells. Then, it disturbs the substrate binding and interferes with wild-type CHEK2 in a dominant-negative manner (Bayram et al. 2012; Bogdanova et al. 2005).

Due to lack of such study in Iranian women population, this preliminary case-control study was conducted to examine the frequencies of four CHEK2 mutations (c.1100delC, del5395bp, IVS2 + 1G > A, and I157T) in BC patients and healthy controls and also to investigate the role of these mutations in susceptibility to BC among Iranian women.

## Materials and method

**Fig. 1:**
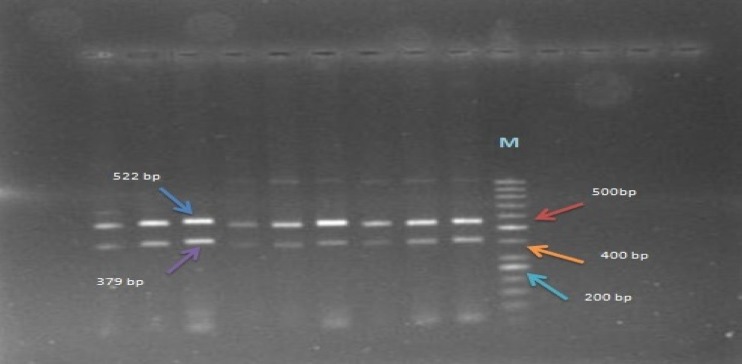
Homozygous wild-type: 522-bp and 379-bp fragment; *M* DNA marker 50 bp

**Fig. 2:**
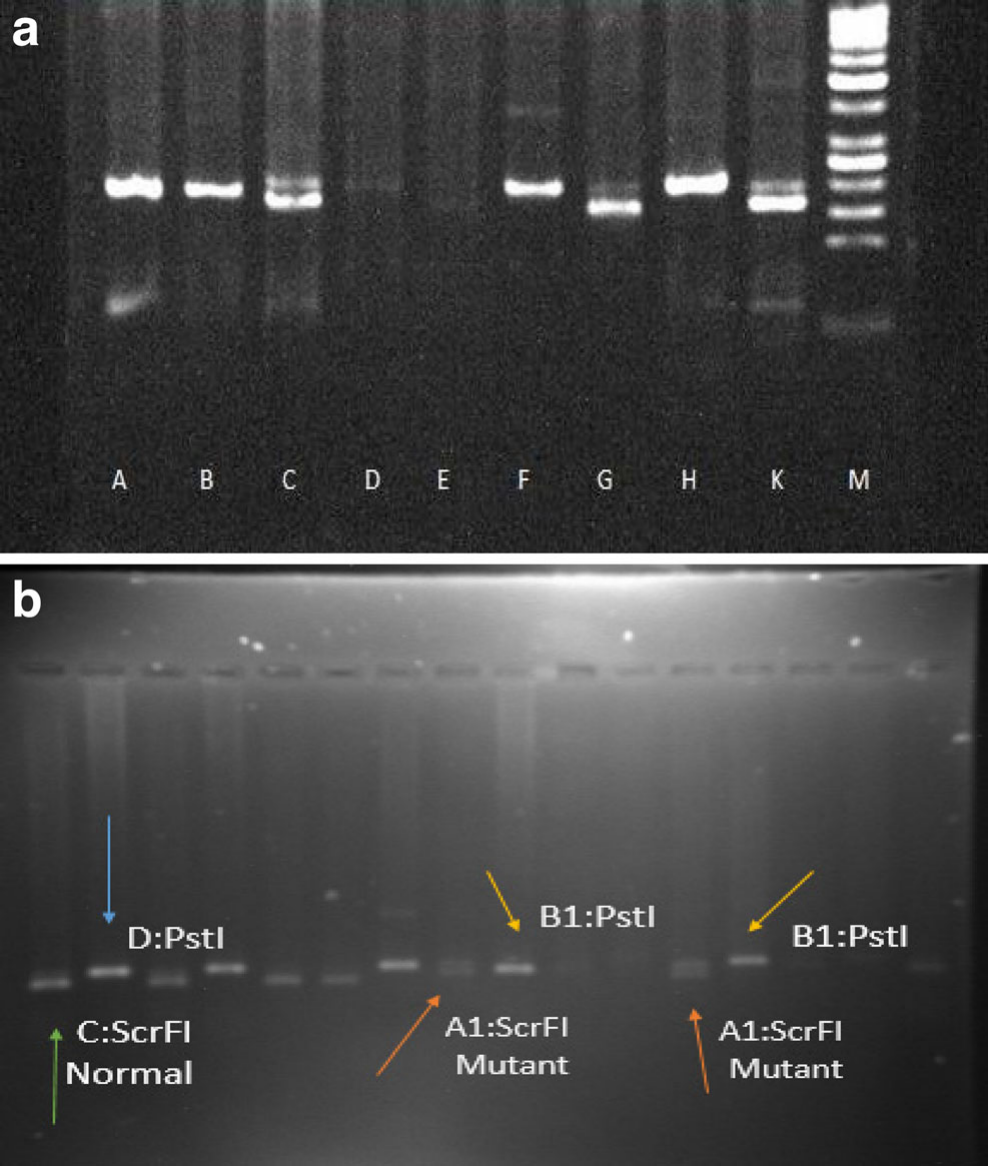
**a** *A* normal DNA, *B* and *H* PCR product uncut with Pst1, *C* and *K* heterozygous mutant-type cut with Scrf1: 194 bp and 174 bp fragmented and *E* negative control (water) *M* DNA marker. **b** Heterozygous mutant-type: 194 bp and 174 bp fragment by screening of PCR products using restriction enzymes ScrfI and PstI; *A1*, heterozygous patient; *B1*, *C*, and *D* homozygous normal

In this study, we examined 100 women with BC for whom the pathological data were confirmed by a pathologist. Other information including age (Table 1), staging (Fig. 1), grading (Fig. 2), and histopathology results (Table 2) of cancer were obtained from the medical records of previous survivors. Lack of breast cancer in normal healthy controls was approved by mammography. Five milliliters of peripheral blood was collected from each BC patient and healthy control and was transferred into a test tube containing EDTA. DNA was extracted from blood samples using a commercial DNA extraction kit (Qiagen, USA). The extracted DNA samples were stored at −80 °C until use. This study was approved by the local ethics committee of Hormozgan University of Medical Sciences, Cellular and Molecular Research Center, Qazvin University of Medical Sciences and Department of Molecular Biology, Pasture Institute of Iran and written informed consent were obtained from all patients and normal controls.

**Table 1:** Descriptive characteristics of individuals with breast cancer and healthy controls

	Breast cancer Mean ± SD	Healthy controls Mean ± SD
Age (year)	48.29 ± 9.39	48.41 ± 8.01
Children number	3.37 ± 1.95	3.54 ± 1.70
Married status	96%	95%
Single status	4%	5%
Women with familial breast cancer	58%	32%
Women with sporadic breast cancer	42%	68%

**Table 2:** Pathologic clinical findings in 100 patients with breast cancer

	Breast cancer after age 45		Breast cancer before age 45	
Variable				
Tumor size <2 cm	29		60	
Tumor size >2 cm	9		8	
	Frequency (%)	*P* value	Frequency (%)	*P* value
Histopathology variables of BC patient				
IDC	34 (%89.4)	0.56	39(%62.9)	0.63
NOS IDC	3 (%7.8)	0.13	20(%32.3)	0.60
ILC	1 (%2.6)	0.58	3(%4.8)	0.54
Grade				
I	2(%5.3)	0.53	2(%3.2)	0.58
II	11(%28.9)	0.65	11(%17.7)	0.73
III	25(%65.8)	0.80	49(%79.30	0.88
Stage				
Ia	6(%15.7)	0.87	9(%14.6)	0.87
Ib	0	0	2(%3.2)	0.50
IIa	17(%44.7)	0.87	33(%53.2)	0.93
IIb	9(%23.6)	0.30	4(%6.5)	0.35
IIIa	3(%7.8)	0.44	8(%12.9)	0.93
IIIb	1(%2.6)	0.69	2(%3.2)	0.63
IIIc	2(%5.2)	0.53	2(%3.2)	0.58
IVa	0	0	2(%3.2)	0.50
ER				
+	19(%50)	0.98	34(%54)	0.99
−	19(%50)	0.98	28(%46)	0.99
PR				
+	15(%39)	0.79	31(%50)	0.87
−	23(%61)	0.83	31(%50)	0.88
Ki67				
+	26(%68)	0.92	11(%17)	0.97
−	12(%32)	0.98	51(%83)	0.97
Her2/neu				
+	5(%13)	0.83	31(%50)	0.90
−	33(%87)	0.63	31(%50)	0.71

## Mutation analyses

All samples from BC patients and healthy controls were tested for c.1100delC, del5395bp, IVS2 + 1G > A, and I157T mutations. All reactions were performed using Veriti Thermal Cycler ABI (Applied Biosystems, Foster City, CA, USA).

### Analysis of CHK2 5395-bp deletion with multiplex-PCR

Multiplex-PCR was also performed for genotyping of large deletion in exon 9 and 10 of CHEK2 gene, as described previously (Cybulski et al. 2007). Multiplex-PCR reaction was performed using specific primers, including the first pair F: 5′- TGTAAT GAG CTG AGA TTG TGC -3′; R: 5′- CAG AAA TGA GAC AGG AAG TT-3′ part breakpoint site in intron 8 and the second pair 5′- GTC TCA AAC TTG GCT GCG -3′; 5′- CTC TGT TGT GTA CAA GTG AC-3′ part breakpoint site in intron 10. In mutation-negative cases, two PCR fragments of 379 and 522 bp were amplified from the wild-type allele. In mutation-positive cases, PCR product of 450 bp was enlarged with the forward primer of the first pair and the reverse primer of the second pair.

Optimal PCR conditions were as follows: a reaction volume of 25 μL containing 2.5 μL 10× buffer (Gen Fanavaran Co), 0.8 μL dNTPs (10 Mm),1.5 μL MgCl2 (50 Mm), 0.3 Taq DNA polymerase (unit/μl), 5 μL of each forward and reverse primers, 5 μL of DNA (20–50 ng/μl), and a remaining volume (16.9 μL) of distilled water (DW). After an initial 10 min at 94 °C, DNA was amplified by 29 cycles of 25 s at 94 °C, 40 s at annealing temperature of 58 °C, and 45 s at 72 °C followed by 1 cycle of 5 min at 72 °C. The presence of PCR products was checked in each reaction by electrophoresis in 1.5% agarose gel followed by visualization step by Gel Red™in gel documentation systems shown in Fig. 1.

### Analysis of CHK2 IVS2 + 1G > A and I157T mutations with PCR-RFLP

CHEK2 IVS2 + 1G > A mutation was examined using PCR-RFLP as explained previously (Bogdanova et al. 2005). A genomic region including both the IVS2 + 1G > A and I157T mutations in intron2 and exon3 of the CHEK2 gene was amplified by PCR using mutagenic primers to allow the restriction enzyme to examine the occurrence of these two mutations.

The 194-bp fragment surrounds the G to A frame shift mutation site in CHK2 IVS2 + 1G > A splice site in intron 2 and the T to C substitution mutation in CHK2 I157T site of exon3. PCR was performed using specific primers F: 5′- GCAAGAAACACTTTCGGATTTTCCGG -3′ and R: 5′-CCACTGTGATCTTCTATGTCTGCA-3′. Optimal PCR conditions were as follows: a reaction volume of 25 μL containing 2.5 μL 10X buffer (Gen Fanavaran Co), 0.8 μL dNTPs (10 Mm), 1.5 μL MgCl2 (50 Mm), 0.3 Taq DNA polymerase (unit/μl), 5 μL of each forward and reverse primers, 5 μL of DNA (20-50 ng/μl), and a remaining volume (13.9 μL) of distilled water (DW).

After an initial 5 min at 95 °C, DNA was amplified by 33 cycles of 45 s at 94 °C, 40 s at annealing temperature of 61.5 °C, and 45 s at 72 °C followed by 1 cycle of 5 min at 72 °C. PCR products were separately incubated for 16 h with either ScrFI or PstI (New England Bio labs, Beverly, MA). Restriction enzyme reaction products were separated on a 3% agarose gel and visualized by gel red (Gel Red, UK) in a gel documentation system.

To evaluate I157T mutation, the 194-bp product was cleaved by PstI into two fragments of 20 and 170 bp, while the normal product was not cleaved. In case of IVS211G > A mutation, the normal product was cleaved by ScrFI and the mutant PCR product was not cleaved. All positive cases were confirmed by direct sequencing of PCR products using the intronic primers 5′-CCTTCTTAGGCTATTTTCCTAC-3′ (forward) and 5′-AACCATATTCTGTAAGGACAGG-3′ (reverse). Sequencing was accomplished using the forward primer and the sequences were assessed on Genetic Analyzer 3130 (Applied Biosystems, Hitachi, USA). The results of this assay are shown in Fig. 2a, b.

### Analysis of CHEK2 1100delC mutation with AS-PCR

Allele-specific PCR was performed to detect the 1100delC mutation of CHK2 gene as described previously (Rashid 2005). Genotyping of CHEK2 mutation was executed using allele-specific PCR amplification with primers CHEK2 (Jahani & Ghotbi 2002) ex10F:(5′-GCA AAA TTA AAT GTC CTA ACT TGC-3′), CHEK2ex10R:(5′-GGC ATG GTG GTGTGC ATC-3′) and CHEK2delC:(5′-TGG AGT GCC CAA AAT CATA-3′). In mutation-negative cases, the PCR product of 537 bp was enlarged from the wild-type allele and PCR product of 200 bp was amplified in mutation-positive cases.

**Fig. 3:**
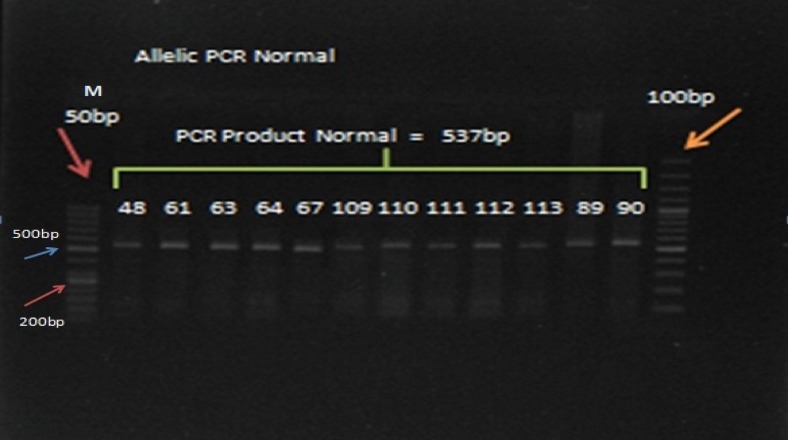
Homozygous wild-type: 537-bp fragment; *M* DNA marker 50 bp and 100 bp

Optimal PCR conditions were as follows: a reaction volume of 25 μL containing 2.5 μL 10× buffer (Gen Fanavaran Co), 0.8 μL dNTPs (10 Mm), 1.5 μL MgCl2 (50 Mm), 0.2 Taq DNA polymerase (unit/μl), 0.5 μL of each forward and reverse primer, 1 μL of DNA (20–50 ng/μL), and distilled water (18 μL). After an initial 10 min at 94 °C, DNA was amplified by 29 cycles of 25 s at 94 °C, 30 s at annealing temperature of 52 °C, and 35 s at 72 °C followed by 1 cycle of 5 min at 72 °C. PCR products were electrophoresed on 1.5% agarose gel containing 0.5 Gel red (Gel Red, UK) and visualized in a gel documentation system (Gel Logic 212 PRO, USA), as illustrated in Fig. 3.

### Statistical analysis

The prevalence of three CHEK2 alleles in cases and controls was assessed between variables using SPSS17.0 software and EpiInfo 3.5.4 test. Odds ratios were generated from two-by-two tables using EpiInfo 3.5.4. *p* ≤ 0.05 was considered as significant. All mutations were within the anticipated allocation according to Hardy-Weinberg equilibrium in both groups.

## Results

We examined the presence of four mutations in CHEK2 gene in 100 breast cancer patients with a personal and/or family history of breast cancer. The mean age of patients was 48.29 ± 9.39 years (range: 26–76). Controls were healthy females with similar age range 48.41 ± 8.01 (range 26–80) (Table 1). Among the patients, 96% were diagnosed with invasive ductal carcinoma (IDC) from whom 73% were IDC, 23% IDC (NOS: not otherwise specified) and the rest with invasive lobular carcinoma (ILC) (Table 2).

**Fig. 4:**
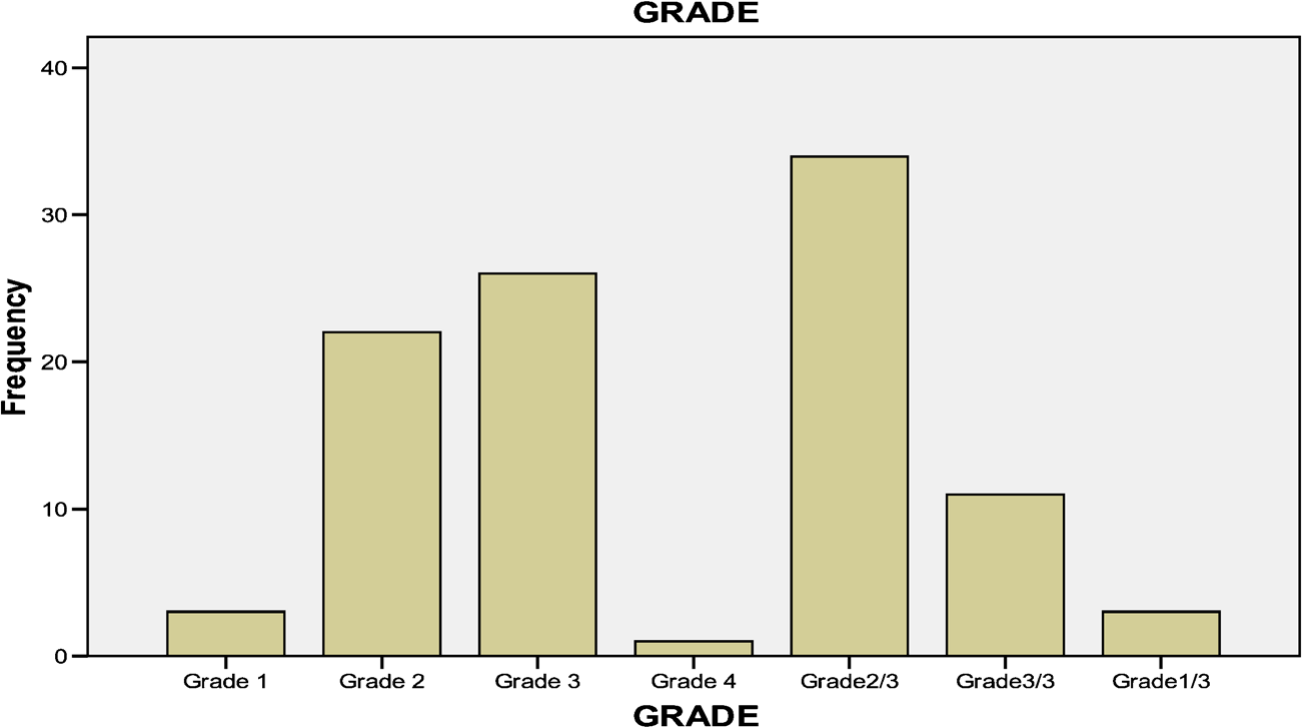
Percentage of different grades of tumor patients

**Fig. 5:**
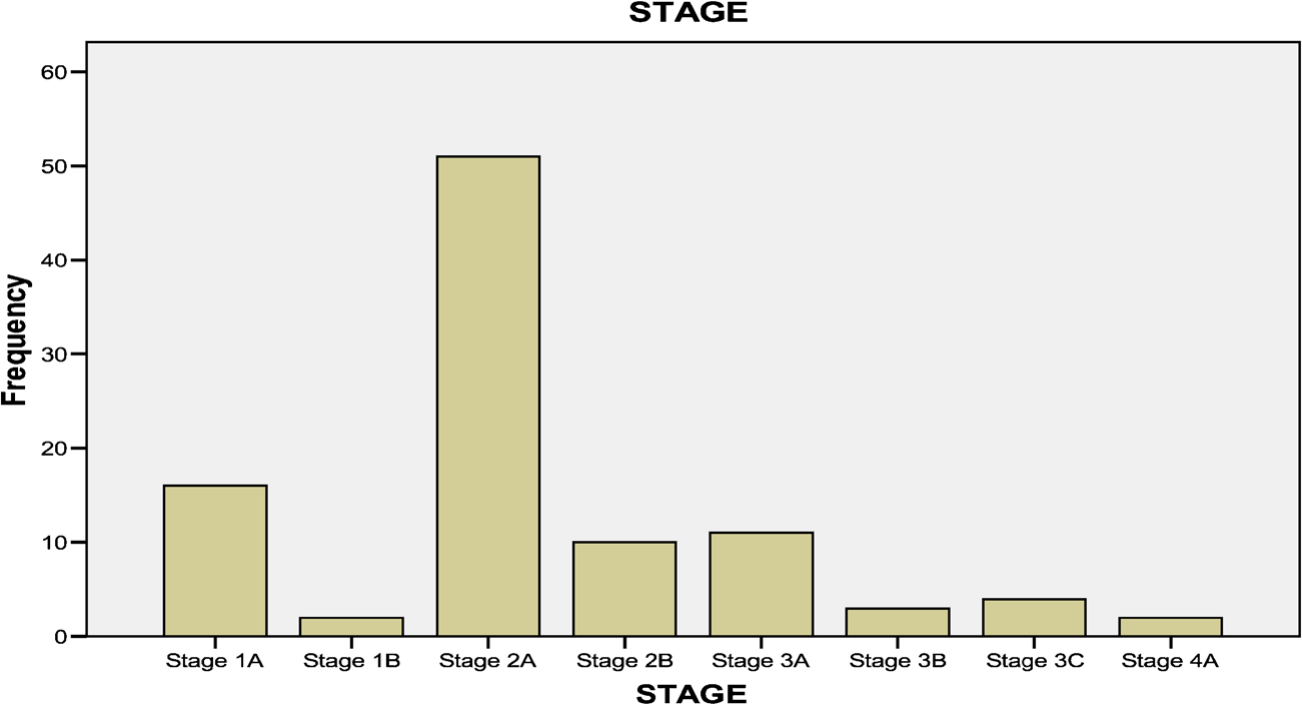
Frequency of different stages of tumor patients

In total, 6% of patients were diagnosed with grade I, 56% with grade II, 37% with grade III, and 1% with grade IV, respectively (Fig. 4). The most common tumor stage was IIA (Fig. 5 and Table 2).

Ninety-six percent of patients were married and 4% were single (Table 1). Fifty-eight percent of patients had a family history of breast cancer (Table 1). Thirty-eight percent of patients had an average age of less than 45 years (Table 1) and 10% had bilateral breast cancer (Table 4).

In total, 100 BC cases and 100 controls selected for our study were successfully analyzed for 1100delC, IVS2 + 1G > A, I157T, and del5395bp mutations in CHEK2 gene using AS-PCR, PCR-RFLP, and Multiplex-PCR, respectively. Our observation showed a low frequency of IVS2 + 1G > A mutation in two cases. The missense variant was present in 2% of the cases (*p* = 0.48) as presented in Table 3.

**Table 3:** Frequency of the CHEK2 mutations in Iranian population

Mutation	Case(No = 100)		Control(No = 100)		*P* value
	Yes	No	Yes	No	
With IVS2 + 1G > A	2	98	0	100	0.48
With I157T	0	100	0	100	0
With 5395 bp	0	100	0	100	0
With 1100delC	0	100	0	100	0

A positive case of CHEK2 mutation IVS2 + 1G > A was associated with hereditary breast cancer and the other found to have a sporadic nature (Table 4). None of the 100 analyzed samples carried the CHEK2 1100delC, I157T, and del5395bp mutations (Table 3). These results suggest that these mutations are absent or perhaps present at a really low frequency among Iranian population; nevertheless, we can verify the low risk associated with frame shift variant of IVS2 + 1G > A.

**Table 4:** The relationship between IVS2 + 1G > A mutation and clinic pathology parameter of breast cancer

Characteristic	Overall	Present	Absent	*P* value
>45	62(62%)	2(3.2%)	60(97.3%)	1.00
≤45	38(38%)	0	38(100%)	
Familial breast cancer	58(58%)	1(1.4%)	57(98.5%)	0.25
Unselected breast cancer	42(42%)	1(1.4%)	41(98.5%))	
Bilateral breast cancer	10(10%)	0	10(100%)	
Histological type				
IDC	73(74%)	1(1.5%)	72(98.5%)	0.6
IDC (NOS)	23(23%)	1(4%)	22(95.6%)	
ILC	4(3%)	0	4(100%)	
Stage				
1A	16(16%)	1(6.2%)	15(93.7%)	0.1
2A	52(52%)	1(1.9%)	51(98%)	

## Discussion

Forthe first time,we evaluated the incidence of CHEK2 mutations in a case-control study of BC among Iranian women. Unfortunately, only 30% of BC risk factors are known and the additional causes of most cases are unknown (Dolan & Glasser 2000 **).** BRCA1 and BRCA2 susceptibility genes have been identified in BC in previous studies (Miki et al. 1994; Wooster et al. 1995). In addition, the genes CHEK2, ATM, PALB2 **(the partner and localizer of the BRCA2 gene)** BRIP **(BRCA1-interactingprotein gene**), and NCOA3 **(Nuclear Receptor Coactivator 3)** are considered as predisposing genes increasingthe risk of BC (Jahani & Ghotbi 2002). Therefore, we analyzed any CHEK2 mutation as well asassociation between these mutations and breast cancer in Iranian women.

Initially, we analyzed these mutations in an equal number (100) of breast cancer and control cases. We found IVS2 + 1G > A missense variant in a positive case of CHEK2 mutation IVS2 + 1G > A, which was associated with hereditary breast cancer and the other that was found to have a sporadic nature (2 out of 100 patients) (*p* = 0.48). By examining the results of Hardy-Weinberg equilibrium using chi-square test, our results have no statistically significant relationship (*p* = 0/48) in our society.

We showed that this mutation was present with a very low frequency in breast cancer patients and healthy controls of the Iranian population. Similar results have reported that the CHEK2 IVS2 + 1G > A variants are associated with BC risk (Bogdanova et al. 2005; Consortium 2004). None of these 100 samples had CHEK2 1100delC (Sodha et al. 2006), I157T (Strachan & Read 1996), and del5395bp mutations. Our results are similar to those reporting that there is no association between CHEK2 1100delC (Rashid 2005; Ndawula et al. 2014), I157T (Kilpivaara et al. 2004; Bayram et al. 2012), and del5395bp (Mohelnikova-Duchonova et al. 2010; Cybulski et al. 2007) variations and breast cancer risk and our cases were not selected based on family history.

CHK2 IVS2 + 1G > A mutation has a lower geographic distribution (Cybulski et al. 2007; Bogdanova et al. 2005) whereas the I157T mutation shows a higher geographic distribution (Cybulski et al. 2004a; Cybulski et al. 2004b). This variant has been reported in ethnically diverse populations and is associated with a modest risk for developing BC among German and Belarusian populations (Bogdanova et al. 2005). Also, the protein-truncating variant IVS2 + 1G > A mutation is detected in Slavic populations of Eastern Europe, German, and Byelorussian populations (Cybulski et al. 2007; Bogdanova et al. 2007)as well as Polish cancer patients (Cybulski et al. 2004b).

There are only few studies in some countries investigating the possible relationship between IVS2 + 1G > A CHEK2 gene mutation and an increased risk of breast cancer (Bogdanova et al. 2005). It should be noted that the rare occurrence of IVS2 + 1G > A mutation may be related to lack of sufficient studies reported from different geographical regions (Einarsdóttir et al. 2006).

These results confirm the geographical and ethnic differences between populations and the need for further investigations in different nations.

Although it is widely accepted that the risk of BC may be higher for women who have both a CHEK2 gene mutation and a family history of BC (Cybulski et al. 2011), our results fail to clearly demonstrate a role for CHEK2 mutation IVS2 + 1G > A in inherited susceptibility to breast cancer similar to other studies (Bogdanova et al. 2005; Liu et al., 2012).This may be explained by an interaction of CHEK2 mutations with susceptibility alleles in other genes to increase the inherited BC prevalence (Kilpivaara et al. 2004; Consortium 2004).

The I157T mutation has not been identified in different populations (Consortium 2004; Einarsdóttir et al. 2006). Moreover, no increased risk of breast cancer due to I157T CHEK2 gene mutation was observed in Moroccan population (ElAmrani et al. 2014). I157T had a lower incidence in some countries when compared to 1100delC mutation in patients with breast cancer (Consortium 2004; Meijers-Heijboer et al. 2003).

We did not find the 1100delC mutation in Iranian population, which is in line with previous studies and led us to propose that the c.1100delC may not contribute to BC susceptibility in Asian (Choi et al. 2008; Song et al. 2006; Bell et al. 2007; Lee & Ang 2008; Rajkumar et al. 2003) countries and North America (González-Hormazábal et al. 2008), compared to the pattern observed in Northern and Eastern European countries (ElAmrani et al., 2014). On the other hand, these countries have a traditionally common origin compared to other countries.

These findings are in agreement with the hypothesis that the existence of a c.1100delC frequency gradient from these regions is caused by an ancestrally common origin in the North (ElAmrani et al., 2014). This gradient may be more accentuated in the Middle East countries, which may explain the absence of this mutation among Iranian population.

CHEK2 del5395bp gene mutation increases the risk of breast cancer in some countries (Cybulski et al., 2007; Bąk et al., 2014).

Both protein-truncating mutations (CHK2 1100delC, I157T, and IVS2 + 1G > A) are reported to be associated with breast, prostate, thyroid, kidney, and bladder cancer (Cybulski et al., 2004b). In contrast, several studies have shown no association between these mutations and susceptibility to cancer (González-Hormazábal et al., 2008; Osorio et al., 2004). Interestingly, two different investigators have reported that the CHEK2 I157Tmutation seems to be protective against lung cancer in patients from Eastern Europe (Cybulski et al., 2008).

Obviously, a similar type of study with a higher number of samples could be useful to show the possible increase in frequency of CHEK2 mutation and may lead to faster diagnosis of patients with breast cancer. On the basis of results obtained from different countries with bigger samples, the association between increased risk of breast cancer and CHEK2 gene mutation has been confirmed.

Several reasons could explain this situation. Firstly, the number of individuals recruited in this study was comparatively small and some relations may have been missed as a result of limited study. Secondly, it was a hospital-based investigation and the study populations were selected from a single organization (Tehran University, Milad Hospital); therefore, the selection bias was inevitable, and the participants may not have presented the common ethnic characteristics of the whole Iranian society. Thirdly, the sample size used in our study was preferred according to several previous studies (ElAmrani et al. 2014; Meijers-Heijboer et al. 2003; Choi et al. 2008; Song et al. 2006; Bell et al. 2007; Lee & Ang 2008; Rajkumar et al. 2003; González-Hormazábal et al. 2008; Bąk et al. 2014; Osorio et al. 2004; Cybulski et al. 2008; Iniesta et al. 2010), and it is therefore essential for our current findings to be confirmed in a larger independent study. Fourthly, the present study only focused on a single gene with no considerations on gene-environment and gene-gene interactions, which can influence the characteristic susceptibility to BC.

In conclusion, for the first time, we identified one out of four different CHEK2 alterations in two patients (2%) and the occurrence of 1100delC, I157T, and del5395bp mutations in CHEK2 gene, which are usually absent or are present at really low frequency in breast cancer patients and healthy controls of the Iranian population.

As a result, we concluded that it is not a suitable predictive test for other CHEK2 mutations in a clinical setting for breast cancer among Iranian population. On the other hand, further studies examining the total coding sequence of CHEK2 must be performed. Our study reveals this relationship, and although the number of patients was low, the patients and controls were fully age-matched.

Moreover, many large-scale studies are needed to confirm our results, particularly in patients with different ethnic origins for better understanding of CHEK2 1100delC, IVS2 + 1G > A, del5395bp, and I157T mutations and susceptibility to breast cancer. However, the overall number of detected variants in our study was relatively small, and a number of associations may have been missed as a result of limited study scale. The authors suggest further studies regarding the gene-gene interaction between CHEK2 gene and other tumor suppressor genes to demonstrate the cancer risk in Iranian women. Finally, in agreement with previous studies, except for IVS2 + 1G > A mutation (which is usually observed rarely), the other three CHEK2 mutations do not play an important role in the breast cancer risk in Iran
